# Efficacy of durvalumab plus chemotherapy in small-cell lung cancer with Lambert-Eaton myasthenic syndrome

**DOI:** 10.1016/j.rmcr.2023.101974

**Published:** 2023-12-23

**Authors:** Naoya Ishibashi, Toshiharu Tabata, Ryo Nonomura, Yutaka Oshima, Takanobu Sasaki, Hideki Mitomo, Takafumi Sugawara, Motoyasu Sagawa

**Affiliations:** Department of Thoracic Surgery, Tohoku Medical and Pharmaceutical University Hospital, Japan

**Keywords:** Durvalumab, Small-cell lung cancer (SCLC), Lambert-Eaton myasthenic syndrome (LEMS)

## Abstract

Lambert-Eaton myasthenic syndrome (LEMS) is a rare disease but is often associated with small-cell lung cancer (SCLC). We discuss the case of a 65-year-old man diagnosed with SCLC-LEMS and treated with carboplatin, etoposide, and durvalumab. Lower extremity weakness and high anti-P/Q voltage-gated calcium channel (VGCC) antibody levels were diagnostic and helpful.

The patient showed a reduction in neurological symptoms with treatment for SCLC, including an immune checkpoint inhibitor (ICI), without standard treatment for LEMS. This treatment may be a treatment option, although the recurrence of LEMS as an immune-related adverse events (irAEs) should be noted.

## 1 Introduction

Lambert-Eaton myasthenic syndrome (LEMS) is a rare disease characterized by myasthenic symptoms mainly in the lower extremities caused by autoantibodies. LEMS has also been associated with malignant diseases and is often associated with small-cell lung cancer. In this study, we experienced a case of LEMS associated with small-cell lung cancer in which the neurological symptoms improved due to the response to chemoimmunotherapy without 3,4-diaminopyridine.

### 1.1 Case presentation

**Fig. 1:**
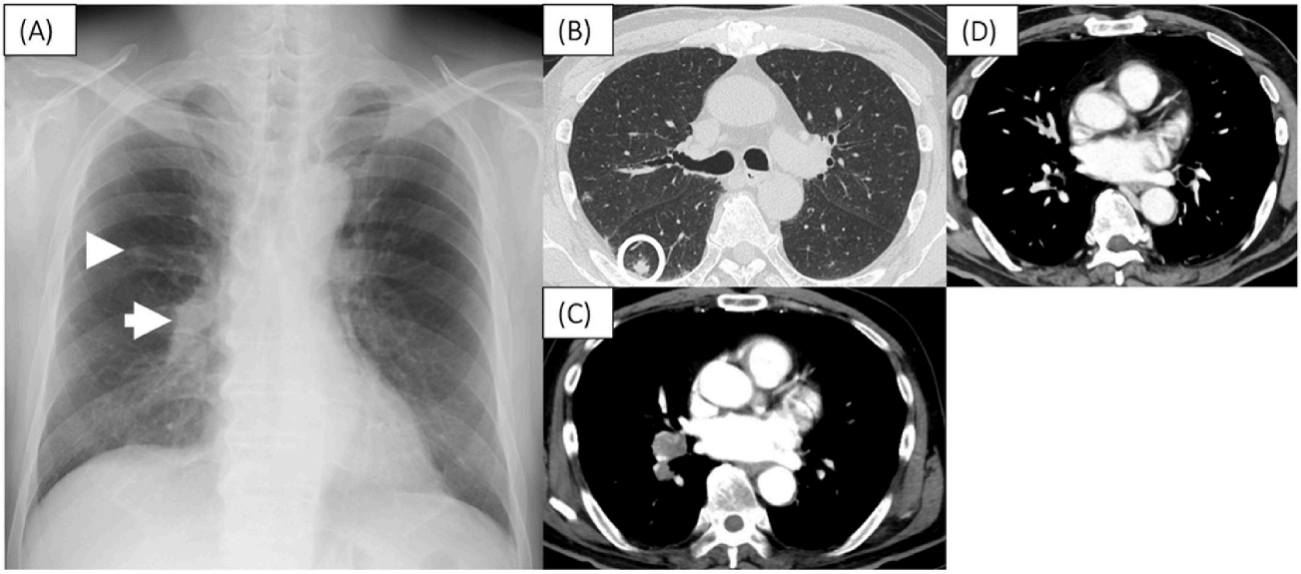
Chest X-ray film on admission showed tumor shadows in the right middle lung field (arrowhead) and right hilar hilum swelling (arrow) (A). Chest CT on admission. CT showed an 11mm mass in the right lower lobe (circle) (B). Contrast-enhanced CT showed multiple right hilar hilum lymph swelling (C). CT after 6 courses of durvalumab showed marked reduction of hilar lymph nodes (D).

A 65-year-old Japanese male patient noticed fatigue and muscle weakness in both lower limbs, which worsened over time, and presented to our neurology department one month later. Contrast-enhanced magnetic resonance imaging (MRI) of the head showed no abnormal findings, including in the cerebellum. The neurological examination showed no cerebellar ataxia symptoms, but muscle weakness was observed in the proximal muscles of both lower limbs. A simple chest radiograph showed a nodular shadow in the right middle lung area (Fig. 1A), and a chest computed tomography (CT) scan showed a tumor with a diameter of 11 mm in the right lower lung and enlarged hilar lymph nodes (Fig. 1B and C).

**Fig. 2:**
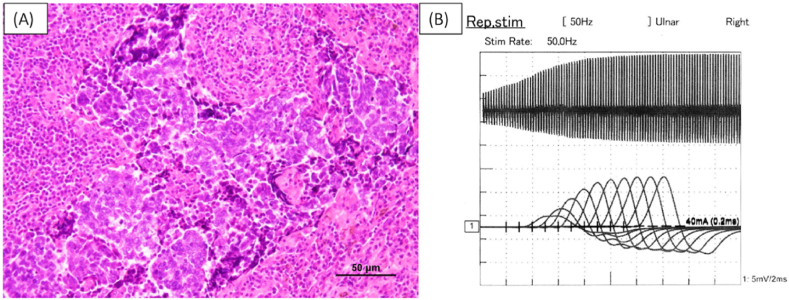
Histological findings from left lung partial resection reveal small cell carcinoma ((A). H.E. stain). Waxing was observed with 50Hz stimulation of the ulnar nerve (B).

Blood tests showed high serum Pro-gastrin-releasing peptide (ProGRP) of 140 pg/mL (normal value < 81 pg/mL) and anti-P/Q-type voltage-gated calcium channel (P/Q VGCC) antibody of more than 250 pmol/L (normal value < 30 pmol/L), and Lambert-Eaton myasthenia syndrome (LEMS) due to small-cell lung cancer (SCLC) was suspected, and the patient was referred to our department. A partial resection of the right lower lung was performed, and the diagnosis of SCLC was confirmed (Fig. 2A). Postoperative repetitive stimulation-evoked electromyography of the ulnar nerve showed a waxing phenomenon at 50 Hz (Fig. 2B).

Based on the above, a diagnosis of LEMS due to SCLC was made. After surgery, he received systemic chemoimmunotherapy with carboplatin (area under the curve 5mg/mL/min, day 1), etoposide (80 mg/m2, day 1–3), and durvalumab (1500 mg/body, day 1). After two courses, myasthenia of both lower limbs gradually recovered and became no longer a problem in daily life. After two more courses of the same regimen as above, maintenance therapy with durvalumab was continued on an outpatient basis, and CT showed reduction of hilar lymph nodes (Fig. 1D). Additionally, muscle weakness on the proximal side of both lower extremities has not flared up. Serum ProGRP levels remain within the normal range, and anti-P/Q VGCC antibodies rose temporarily after the start of chemotherapy but have since declined from predose levels.

## 2 Discussion

LEMS is characterized by abnormalities of the neuromuscular junction. It is a particularly common example of tumor-associated neurological syndrome with a high rate of SCLC. Autoantibodies targeting P/Q VGCC distributed on the surface of cancer cell membranes are produced, and these antibodies decrease the amount of P/Q VGCC at the neuromuscular junction, resulting in lower extremity muscle weakness and autonomic nervous system dysfunction [1,2].

In recent years, combining ICI with cytotoxic chemotherapy has appeared as an approach to expand treatment options for SCLC [3,4]. ICI selectively block the programmed cell death ligand 1 (PDL-1) on cancer cell surfaces and programmed cell death 1 (PD-1) on T lymphocytes. This interruption of inhibitory signals to T lymphocytes maintains cancer cell activation and exerts anti-cancer effects. However, this therapeutic strategy has the potential to develop irAEs such as myasthenia gravis and LEMS as neuromuscular junction disorders. Consequently, ICI is not recommended for patients with autoimmune diseases (AIDs) or a history of AIDs. The effectiveness and safety of ICI in lung cancer patients with concurrent AIDs remain uncertain, particularly for those receiving high-dose steroids or immunosuppressive agents. However, SCLC-LEMS patients with concomitant AIDs disease but whose condition is stable may benefit from ICI therapy [5].

Chalk et al. reported the potential for improving LEMS symptoms by prioritizing non-ICI cancer treatments in 16 patients with SCLC-LEMS [6]. Other reports have also demonstrated the efficacy of ICI in LEMS (Table 1). Dohrn et al. documented the use of avelumab for Merkel cell carcinoma, which led to regression of the primary lesion but worsened neurological symptoms, after relieved with intravenous immunoglobulin (IVIG) [7]. Similarly, Green et al. presented the use of avelumab for Merkel cell carcinoma, showing regression of the primary lesion and improvement of neurological symptoms [8]. Additionally, Takigawa et al. administered pembrolizumab to a patient with squamous cell lung cancer as a secondary cancer with a history of SCLC-LEMS remission. Although the primary lesion decreased in size, LEMS symptoms recurred but were later alleviated with IVIG [9]. These cases collectively suggest the potential efficacy of IVIG in treating LEMS as an irAE. Studies by Sakaguchi and Machiyama using atezolizumab and durvalumab, respectively, in extensive-disease small cell lung cancer (ED-SCLC) have reported the effectiveness of ICI in controlling the primary lesion and improving neurological symptoms. Management of neurological symptoms in these reports involved the use of IVIG, 3,4-diaminopyridine, pyridostigmine, and other medications [10,11]. In cases with severe neurological symptoms, these medications should be considered before starting chemotherapy.

**Table 1:** Literature review of cases of LEMS treatment with ICI.

Case	Author name (year)	Age	Sex	Pathology	LEMS therapy	ICI	Anti-P/Q-type VGCC antibodies		Symptom	Tumor response
							Pre-treatment	Post-Treatment		
1	Dohrn et al. (2020)	62	F	MCC	3,4-diaminopyridine, pyridostigmine, IVIG	Avelumab	387.6	NA	Worsen	CR
2	Sakaguchi et al. (2022)	56	M	SCLC	None	Atezolizumab	>30	NA	Improvement	PR
3	Green et al. (2022)	67	M	MCC	3,4-diaminopyridine, pyridostigmine	Avelumab	65.2	ND	Improvement	PR
4	Machiyama et al. (2023)	62	F	SCLC	pyridostigmine	Durbalumab	1419.2	263.5	Improvement	PR
5	Crrent case	65	M	SCLC	None	Durbalumab	>250	130	Improvement	PR

LEMS: Lambert-Eaton myasthenic syndrome, ICI: Immune checkpoint inhibitor, MCC: Merkel cell carcinoma, SCLC: Small-cell lung carcinoma, IVIG: Intravenous immunoglobulin, NA: Not available, ND: Not detected, CR: Complete response, PR: Partial response.

In this case, the neurological symptoms were mild, so after consultation with the neurologist, treatment of the underlying cause of LEMS, SCLC, was prioritized instead of IVIG or 3,4-diaminopyridine. Symptoms by controlling SCLC with ICI plus chemotherapy. Although chemoimmunotherapy is not recommended for all SCLC-LEMS patients, it should not be ruled out as an option for ICI therapy and may benefit from careful use in patients with mild and stable neurological symptom.

## 3 Conclusion

ICI plus chemotherapy for SCLC-LEMS was effective in controlling cancer progression and improving neurological symptoms. Prudent use of ICI plus chemotherapy can improve the prognosis.

## Consent for publication

Written informed consent for publication was obtained from the patient discussed in this article.

## Funding

This research did not receive any specific grant from funding agencies, commercial, or not-for-profit sectors.

## Declaration of competing interest

The authors declare that they have no known competing financial interests or personal relationships that could have appeared to influence the work reported in this paper.
